# Proteomic analysis of short-term preload-induced eccentric cardiac hypertrophy

**DOI:** 10.1186/s12967-016-0898-5

**Published:** 2016-05-27

**Authors:** Belal A. Mohamed, Abdul R. Asif, Moritz Schnelle, Mohamed Qasim, Sara Khadjeh, Dawid Lbik, Peter Schott, Gerd Hasenfuss, Karl Toischer

**Affiliations:** Department of Cardiology and Pneumology, University Medical Center, Goettingen, Germany; DZHK (German Centre for Cardiovascular Research), Partner Site Goettingen, Germany; Department of Medical Biochemistry and Molecular Biology, Faculty of Medicine, Mansoura University, Mansoura, Egypt; Institute of Clinical Chemistry/UMG-Laboratories, University Medical Center, Goettingen, Germany; Department of Microbiology, Kohat University of Science and Technology, Kohat, Pakistan; Abteilung Kardiologie und Pneumologie, Universitätsmedizin Göttingen, Robert-Koch-Str. 40, 37075 Göttingen, Germany

**Keywords:** Aortocaval shunt, Preload, Eccentric hypertrophy, Heart failure

## Abstract

**Background:**

Hemodynamic load leads to cardiac hypertrophy and heart failure. While afterload (pressure overload) induces concentric hypertrophy, elevation of preload (volume overload) yields eccentric hypertrophy and is associated with a better outcome. Here we analysed the proteomic pattern of mice subjected to short-term preload.

**Methods and Results:**

Female FVB/N mice were subjected to aortocaval shunt-induced volume overload that leads to an eccentric hypertrophy (left ventricular weight/tibia length +31 %) with sustained systolic heart function at 1 week after operation. Two-dimensional gel electrophoresis (2-DE) followed by mass spectrometric analysis showed alteration in the expression of 25 protein spots representing 21 different proteins. 64 % of these protein spots were up-regulated and 36 % of the protein spots were consistently down-regulated. Interestingly, α-1-antitrypsin was down-regulated, indicating higher elastin degradation and possibly contributing to the early dilatation. In addition to contractile and mitochondrial proteins, polymerase I and transcript release factor protein (PTRF) was also up-regulated, possibly contributing to the preload-induced signal transduction.

**Conclusions:**

Our findings reveal the proteomic changes of early-stage eccentric myocardial remodeling after volume overload. Induced expression of some of the respiratory chain enzymes suggests a metabolic shift towards an oxidative phosphorylation that might contribute to the favorable remodeling seen in early VO. Down-regulation of α-1-antitrypsin might contribute to extracellular matrix remodeling and left ventricular dilatation. We also identified PTRF as a potential signaling regulator of volume overload-induced cardiac hypertrophy.

**Electronic supplementary material:**

The online version of this article (doi:10.1186/s12967-016-0898-5) contains supplementary material, which is available to authorized users.

## Background

In the heart, hemodynamic load is a critical regulator of myocardial function, gene expression and phenotype appearance [1]. Thereby two types of load can be differentiated, namely preload and afterload. Preload increases during diastolic filling and passively stretches cardiomyocytes. This results in immediate recruitment of contractile units and increased cardiac performance through the Frank-Starling mechanism. In addition, proteins such as titin and associated molecules are stretched with subsequent effects on myocardial elasticity and gene expression [2]. By electromechanical coupling, systolic force is generated by each cardiomyocyte to produce cardiac stroke work against the vascular resistance (afterload). During ejection preload declines again. Both preload and afterload influence load-dependent ion channels and intracellular ion concentrations [3], which in turn may also influence cardiac function and gene expression. A pathologically increased afterload occurs in patients with aortic stenosis or arterial hypertension, whereas an increase in preload occurs mainly in patients with mitral or aortic regurgitation. Afterload leads to a concentric hypertrophy with an increase in wall thickness and normal left ventricular volume, whereas preload leads to eccentric hypertrophy with normal wall thickness and an increase in the left ventricular volume.

From a hemodynamic point of view, afterload-mediated concentric hypertrophy was long considered beneficial because of stress compensation through increased wall thickness according to the law of Laplace [4]. In contrast, preload-mediated eccentric hypertrophy was considered maladaptive because of uncompensated wall stress. Previously we adjusted two in vivo models of hemodynamic load to compare the effects of mechanical load on the heart failure development. We used the transverse aortic constriction (TAC) as an afterload model and the aortocaval shunt (shunt) as a preload model and could show that preload has a more beneficial phenotype compared to afterload [5]. Afterload leads to an activation of CaMKII, which is involved in the progression of heart failure [6, 7]. Preload is associated with Akt activation without fibrosis, little apoptosis, better function, and lower mortality compared to afterload. Interestingly, B-type natriuretic peptide (*Nppb*), a hallmark for maladaptive remodeling of the LV, was not reactivated up to 7 days in our shunt model [5], which could also be proven in an in vitro model of loaded isolated rabbit muscle strips [8, 9]. Therefore, our current proteome study was performed at the same time point (1 week after shunt) to unravel the potential factors mediating the preload-induced favorable myocardial remodeling.

## Methods

### Animals

The investigation conforms to the Guide for the Care and Use of Laboratory Animals (NIH publication No. 85–23, revised 1996). The experiments were initiated on 8–10 week FVB/N wild-type female animals (Charles River, Sulzfeld, Germany) were used because of high mortality in male mice.

### Aortocaval shunt operation (shunt)

Surgery was performed as described previously [5]. Briefly, mice were anesthetized using isoflurane insufflation. A longitudinal abdominal incision was made and the vessels were prepared. The aorta was clamped and punctured with a needle (23G) through the vena cava inferior. After removing the needle, the external hole in the aorta was closed by cyanoacrylate glue (Pattex, Düsseldorf, Germany). In successful shunt operations mixing of oxygenated blood from the abdominal aorta in the vena cava could be observed. The abdomen was then closed and the mice were kept on a heating plate until full recovery from anesthesia. Sham animals underwent the same procedure except for the puncture of the vessels. At the end of the experiments, mice were intubated, ventilated with 100 % O_2_ and isoflurane. Blood was drawn from the right ventricle and O_2_ saturation was measured. Only animals with right ventricular O_2_ saturation over 90 % were analysed. Hearts were excised and the left ventricle was separated from the atrium and right ventricle. The tissue was shock frozen in liquid nitrogen and stored at −80 °C.

### Histology

The hearts were harvested, fixed in 4 % buffered formaldehyde at 4 °C for 24 h, and cryoprotected in 30 % sucrose/PBS overnight before embedding in OCT (Tissue-Tek). Cardiac sections (8 μm) were stained with haematoxylin-eosin (H&E). Myocyte cross sectional areas were measured, using NIH Image J software (National Institutes of Health, Bethesda, MD, USA), in sections stained with fluorescein conjugated wheat germ agglutinin (WGA- Alexa Fluor 350, Invitrogen, Carlsbad, CA, USA). At least 100 randomly selected myocytes cut transversely were measured from three animals/group. For immunofluorescence staining, cardiac sections were reacted with primary anti-Desmin (1:200; Sigma-Aldrich, Steinheim, Germany) followed by secondary Alexa Fluor 488 IgG antibody (1:500; Invitrogen). DAPI was used for nuclear staining. Sections were analyzed by reverse microscope fluorescence equipped microscope (BX60; Olympus, Hamburg, Germany).

### Echocardiography

2D guided M-mode echocardiography was performed using a VS-VEVO 660/230 high resolution imaging system (VisualSonics, Toronto, Canada). Mice were lightly anesthetized with 2.5 % 2-2-2 Tribromoethanol (Avertin, 0.01 mL/g i.p.) and were allowed to breathe spontaneously. 2D guided M-mode images were recorded in the long-axis view at the left mid-ventricular level. The examiner was blinded towards group assignment.

### Reagents

Reagents and their sources were as follows: phosphate buffer saline (PBS) (PAA Laboratories, Cölbe, Germany), sodium carbonate, ammonium bicarbonate, thiourea, dithiothreitol (DTT), urea, trypsin, trifluoroacetic acid‎ (TFA) (Sigma-Aldrich), CHAPS (AppliChem, Darmstadt, Germany), Acetonitril (ACN) (Promochem, Wesel, Germany), Immobilized pH gradient strips (IPG strips), ampholytes, protein assay kit (Bio-Rad, Munich, Germany), protease and phosphatase inhibitor cocktails (Roche, Mannheim, Germany), sodium dodecyl sulfate (SDS) (Serva, Heidelberg, Germany), potassium ferricynaide, Glycerin, sodium thiosulfate (Merck, Darmstadt, Germany), formic acid (BASF, Ludwigshafen, Germany), bromophenol blue (Carl Roth, Karlsruhe, Germany).

### Sample preparation for proteome analysis

Left ventricular free wall was homogenized and protein extracted as described earlier [10]. Briefly, tissue samples were homogenized in buffer (urea 8 mol/L, thiourea 2 mol/L and 2 % CHAPS) at 2600 rpm for 2 min. Total proteins were extracted by sonication on ice three times for 5 s each with nine cycles in Branson Sonifier 250 (G.Heinemann, Schwäbisch Gmünd, Germany). Whole cell lysates were centrifuged at 16,000×*g* for 15 min at 4 °C. Supernatant was collected in a separate tube and its protein contents was measured by Bradford method using bovine serum albumin as standard and Bio-Rad protein reagent according to manufacturer’s instructions. Tissue proteins were kept at −80 °C until further use.

### Two-dimensional gel electrophoresis (2-DE)

Proteins were resolved by a method adopted from Görg et al. [11] with minor modifications. Proteins (110 µg) from sham and shunt samples were diluted in a final volume of 350 µL with rehydration buffer (7 mol/L urea, 2 mol/L thiourea, 4 % CHAPS, 0.2 % ampholyte [pH 3–10], 0.2 % DTT and trace amount of bromophenol blue). Protein samples were applied to IPG (pH 3–10, Linear) in a rehydration tray overlaid with oil and kept overnight at room temperature in the dark for passive rehydration.

The isoelectric focusing (IEF) was performed using a Protean IEF cell (Bio-Rad) under the following conditions: gradient 100 V for 1 h; 500 V for 1 h; 1000 V for 2 h and 8000 V for 2 h with a total of 32,000 volts-h. Following the first dimensional separation, IPG strips were carefully incubated in equilibration buffer 1 (50 mmol/L Tris–HCL [pH 8.8], 6 mol/L urea, 30 % v/v glycerol, 2 % SDS and 10 g/L DTT) for 30 min and then subsequently for 30 min in equilibration buffer 2 (50 mmol/L Tris–HCL [pH 8.8], 6 mol/L urea, 30 % v/v glycerol, 2 % SDS and 40 g/L iodoacetamide). Proteins were then resolved on 12.5 % SDS-PAGE in Protean II chamber (Bio-Rad) with a constant 100 V at 4 °C.

### Image analysis

Following the second dimensional separation, gels were silver stained by gold standard method, and scanned using a CanoScan 8400F scanner (Canon, Krefeld, Germany). The gel images were densitometrically analyzed with Delta 2D software 4.0 (Decodon, Greifswald, Germany) [12]. LV free walls from 5 shunt hearts were compared with 5 sham controls. Two independent 2-DE experiments were performed to ensure the reproducibility.

### In-gel trypsin digestion of protein spots

The protein spots of interest (differentially regulated proteins) were excised from the gel, which was followed by in-gel digestion according to the method adopted and modified from Shevchenko et al. [13]. In short, gels containing proteins were destained with 100 mmol/L ammonium bicarbonate/acetonitrile (1:1, v/v) initially for 10 min and then until the removal of all dye color. Destaining was followed by drying in vacuum centrifuge (UNIVAPO 150 H; uniEquip, Martinsried, Germany). The proteins in gels were digested with trypsin (10 ng/µL in 100 mmol/L ammonium bicarbonate) for 18 h at 37 °C. Digested peptides were mixed with 50 µL of 0.1 % TFA and sonicated for 30 min. The process was repeated and peptides were extracted with series concentrations of ACN/0.1 % TFA (30 and 60 %). Peptides extracted were concentrated in a vacuum centrifuge and then reconstituted in 0.1 % formic acid.

### Quadruple time-of-flight liquid chromatography/mass spectrometry (Q-TOF LC–MS/MS)

QTOF LC–MS/MS analysis of extracted peptides was performed as described earlier [14]. Briefly, the reconstituted peptide samples (1 μL) were loaded for efficient chromatographic separation on a CapLC-System (Waters, Milford, MA, USA). Peptides were injected for sequence analysis into Q-TOF Ultima Global (Micromass, Manchester, UK) mass spectrometer, equipped with a nanoflow ESI Z-spray source in positive ion mode. MS/MS raw data was generated with MassLynx (Micromass), which was converted to Peak list (pkl) files using ProteinLynx Global Server bioinformatics tool (PLUGS; v 2.2; Waters, Manchester, UK). pkl files were searched by MASCOT search engine against SwissProt data bases for peptide matching. Search criteria includes trypsin enzyme for digestion, mass tolerance of ± 0.5 Da and MS/MS tolerance ± 0.5 Da; allowance of up to one missed cleavage peptide and modification of carbamidomethylation and oxidation of methionine.

### Functional classification

The predicted functions of the differentially expressed proteins were obtained by PANTHER (http://www.pantherdb.org) and UNIPROT knowledge database (http://www.uniprot.org). Differentially expressed proteins were further explored by Ingenuity Pathways Analysis (IPA, Redwood City, CA) (http://www.ingenuity.com) to reveal differentially regulated signaling networks.

### Western blot analysis

Frozen pieces of the left ventricular free wall were thawed on ice in 250 µL of homogenization buffer and homogenized. Protein concentrations of the suspensions were determined and 20 µg of the samples were subjected to SDS-PAGE. Western blotting was carried out according to standard protocols, using antibodies against α-1-antitrypsin, PRDX3, HSPD1, HSPA8, and GAPDH (Santa Cruz Biotechnology, Dallas, USA), COPS4, ß-enolase, MYL2, PTRF, and Ndufs1 (Abcam, Cambridge, UK). For quantification, an enhanced chemiluminescence detection system (Amersham, Braunschweig, Germany) was used according to the manufacturer’s instructions.

### Quantitative real time polymerase chain reaction (qRT-PCR)

DNA-free total RNA was extracted from left myocardial samples by a standard protocol with the RNeasy kit and RNase-free DNAse Set (Qiagen, Hilden, Germany). cDNA synthesis was carried out with iScript cDNA synthesis kit (BioRad, München, Germany) according to manufacturer’s instructions. QRT-PCR was performed on a Biorad iQ-Cycler using SYBR Green Supermix (BioRad). Primer sequences were as follows: *Hprt*: 5′-TCCTCCTCAGACCGCTTTT-3′(sense) and 5′-CATAACCTGGTTCATCATCGC-3′ (antisense); *Cox I*: 5′-TCGAAGGAGTCTCTCGCTCT-3′(sense) and 5′-CTGGTTCTGGCACGGATAGT-3′(antisense); *CoxII*: 5′-CAAGACAGATCATAAGCGAGGA-3′ (sense) and 5′-GGCGCAGTTTATGTTGTCTGT-3′ (antisense); *Atp5a1*: 5′-AAGCTGCAAGGATGCTGTCT-3′ (sense) and 5′-CAACAAAGGATGACCCCAAA-3′ (antisense); *Uqcrc1*: 5′-AGACCCAGGTCAGCATCTTG-3′ (sense) and 5′-CAGCGTCAATCCACACTCC-3′ (antisense).

### Statistical analysis

The echocardiography and Western blotting groups were analysed using unpaired students *t* test, with values of P < 0.05 being considered as statistically significant. Fold change of each protein spot between sham and shunt along with their statistical significance (Student’s *t* test probability) were calculated in the 2-DE gel analyses. Those protein spots having at least 1.5-fold expression changes (P < 0.05) were considered to be statistically significant.

## Results

### Murine model of short-term volume overload-induced eccentric myocardial hypertrophy

**Fig. 1:**
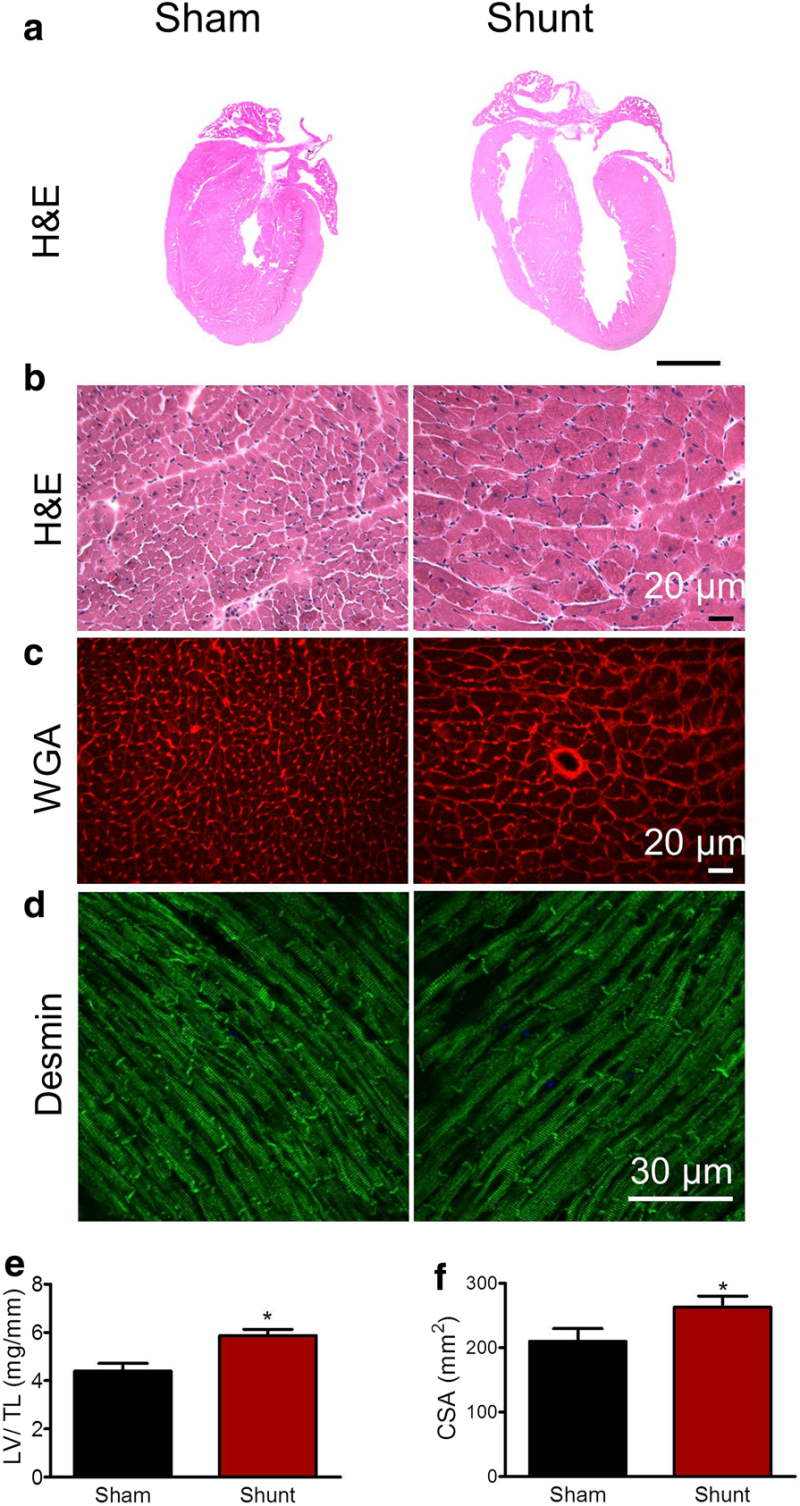
Volume overload-induced myocardial remodeling. **a**–**d** Representative longitudinal sections of hearts (**a**), cardiac trans-sections of the Hematoxylin/eosin (**b**), wheat germ agglutinin (WGA) (**c**), and desmin immunofluorescence (**d**) (*Scale bar* 2 mm for a, 20 µm for **b**–**d**) from sham and shunt mice. **e** Left ventricle/tibial length ratio. **f** Measured cell sectional area (CSA). Values are mean ± SEM; n = 3–5/group. *P < 0.05 vs. sham

Volume overload (VO) was induced in wild-type mice by aortocaval shunt. Seven days after shunt, cardiac hypertrophy was evident by an increased left ventricular (LV) weight to tibia length (TL) ratio by ≈31 % (sham vs. shunt: 4.5 ± 0.2 vs. 5.9 ± 0.2; P < 0.05; Fig. 1e), and an increased cardiomyocyte CSA by ≈25 % (Fig. 1a–c, f). Desmin cytoskeleton was not distorted in shunt hearts as indicated by the maintained striations (Fig. 1d).

**Fig. 2:**
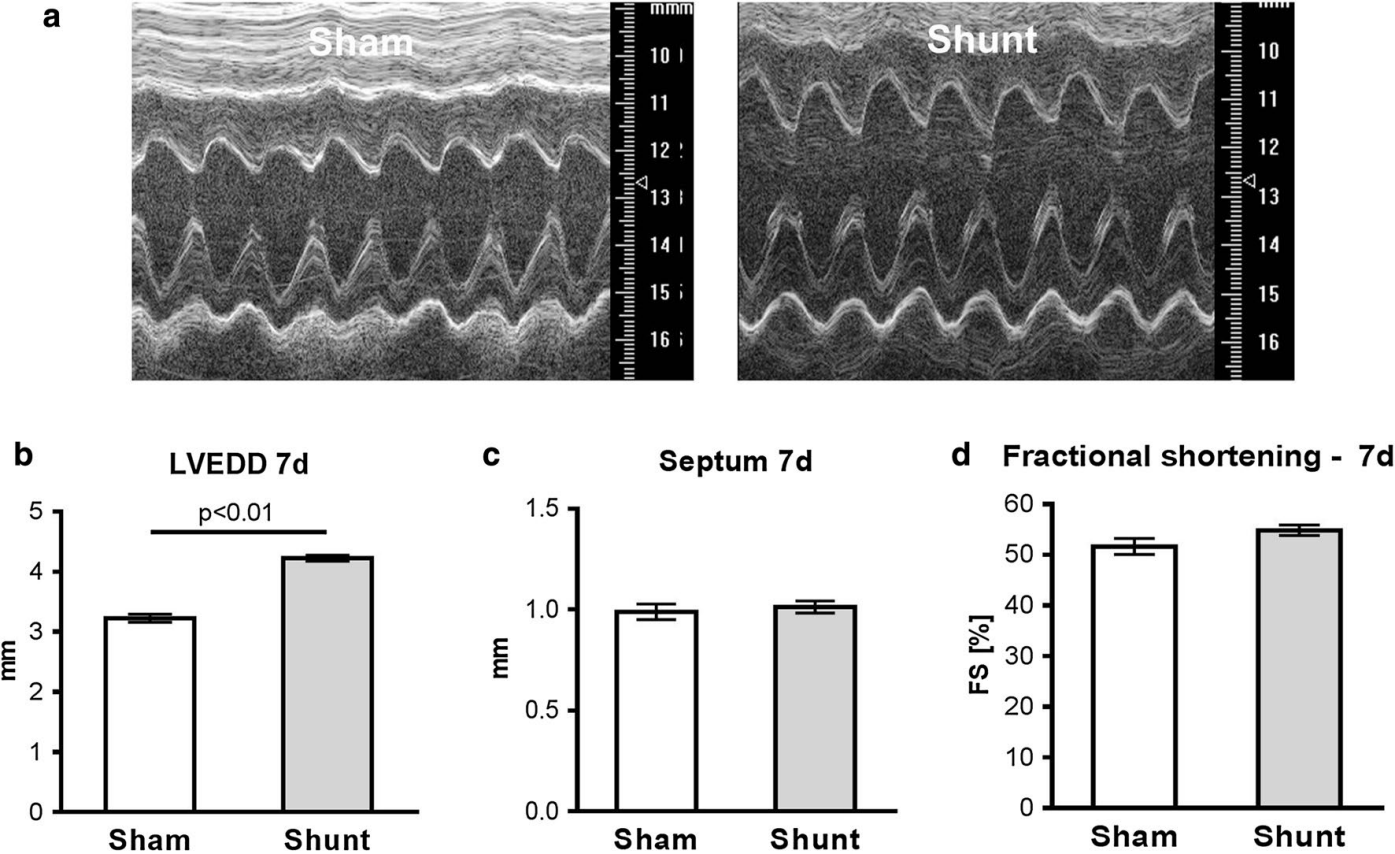
Cardiac structure and function at 1 week of volume overload. **a** Echocardiographic M-mode images. **b**–**d** Left ventricular end-diastolic diameter (LVEDD) (**b**), septum width (**c**), fractional shortening (**d**). Values are mean ± SEM; n = 5/group. *P < 0.05 vs. sham

Representative M-mode echocardiography images demonstrate an increased chamber dimensions observed in shunt mice compared to sham (Fig. 2a). Quantitative analyses showed an eccentric hypertrophy with an increased LV end-diastolic diameter (LVEDD) (sham vs. shunt: 3.23 ± 0.07 vs. 4.23 ± 0.05; +31 %; P < 0.01; Fig. 2b) and unchanged septum wall thickness (sham vs. shunt: 0.98 ± 0.03 vs. 1.01 ± 0.03; P = NS, Fig. 2c). However, the contractile function was preserved (Fractional shortening: sham vs. shunt: 52 ± 2 % vs. 54 ± 1 %, P = NS; Fig. 2d).

### Protein Separation and Q-TOF LC–MS/MS Identification

**Fig. 3:**
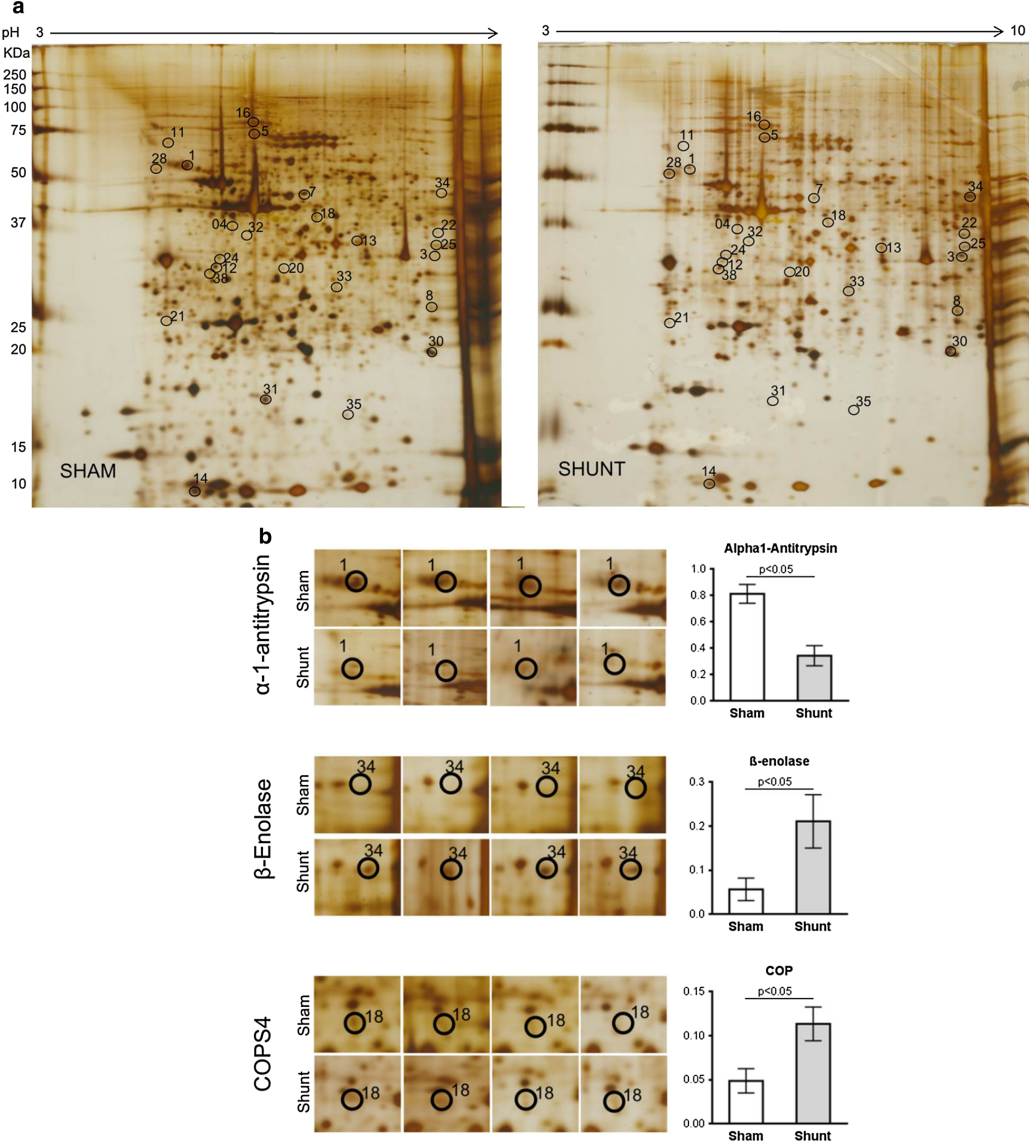
Differentially expressed protein spots in mouse myocardial tissue at 1 week of volume overload. **a** Total protein lysates from sham and shunt myocardium were resolved by 2-DE gel followed by silver staining. *Encircled* differentially expressed protein spots were identified using Q-TOF mass spectrometer. **b** Representative areas in the silver-stained gels show differential expression of α-1-antitrypsin, ß-enolase and COP9 signalosome complex subunit 4 (COPS4) along with their densitometry measurement in sham and shunt. Values are mean ± SEM; n = 5/group; analysis per heart in duplicate

Extracts of cardiac lysates were resolved by 2-DE and then stained with silver staining. Twenty five differentially regulated protein spots were clearly and reproducibly resolved when multiple independent samples from sham and shunt were subjected to analysis (Fig. 3a). The 2-DE expressions of three proteins, α-1-antitrypsin, ß-enolase and COP9 signalosome complex subunit 4 (COPS4) are shown in gel spot pictures (Fig. 3b).

We used Q-TOF LC–MS/MS to identify each protein. The 25 protein spots, representing 21 different proteins, were then analyzed for differential protein expression. The results of these analyses are shown in Table 1.

**Table 1:** Molecular functions of differentially expressed proteins spots in shunt compared to sham

Protein	Spot number	Average sham	Average shunt	T test	Regulation (%)
Cytoskeletal and contractile proteins					
Actin, aortic smooth muscle	5	0266	0126	0007	−53
Actin, cytoplasmic 1	22	0085	0188	0030	121
Myosin-6	20	0010	0026	0029	167
Myosin regulatory light chain 2, ventricular/cardiac muscle isoform	14	0743	0347	0021	−53
Tropomyosin alpha-1 chain	21	0037	0097	0030	162
Tubulin beta-2C chain	4	0010	0026	0006	166
Metabolism					
β-enolase	3	0052	0187	0005	263
β-enolase	34	0057	0211	0047	271
Delta-aminolevulinic acid dehydratase	25	0018	0121	0037	571
L-lactate dehydrogenase B chain	13	0059	0028	0018	−52
Mitochondrial proteins					
ATP synthase subunit alpha, mitochondrial (Complex V)	30	0297	0618	0041	108
ATP synthase subunit d, mitochondrial (Complex V)	31	0266	0068	0042	−74
ATP synthase subunit alpha, mitochondrial (Complex V)	33	0045	0099	0047	119
ATP synthase subunit beta (Complex V)	12	0069	0147	0023	115
ATP synthase subunit beta, mitochondrial (Complex V)	38	0044	0086	0018	96
Cytochrome b-c1 complex subunit 1 (Complex III)	7	0089	0027	0012	−69
Cytochrome b-c1 complex subunit 1, mitochondrial (Complex III)	32	0005	0025	0043	368
Electron transfer flavoprotein subunit beta	8	0094	0249	0015	164
60 kDa heat shock protein, mitochondrial	24	0041	0081	0034	97
NADH-ubiquinone oxidoreductase 75 kDa subunit, mitochondrial	16	0146	0071	0025	−51
Thioredoxin-dependent peroxide reductase, mitochondrial	35	0016	0004	0048	−74
Others					
α-1-antitrypsin 1-2	1	0813	0345	0002	−58
COP9 signalosome complex subunit 4	18	0049	0113	0026	132
Heat shock cognate 71 kDa protein	11	0061	0013	0039	−79
Polymerase I and transcript release factor	28	0304	0657	0039	116

### Proteome analysis

Among these 25 differentially regulated proteins spots, 16 proteins (64 %) were up-regulated in shunt-operated mice, ranging from 2- to 4.7-fold increases. The remaining 9 protein spots (36 %) were down-regulated in shunt-operated mice with levels that were 2.1–4.8-fold lower than in the sham hearts (Table 1). Additional file 1: Table S1 shows the pI, molecular masses, Mascot score, SwissProt accession numbers and MS/MS spectral information.

To better understand the molecular functions and predict the subcellular localizations of the differentially expressed proteins, we searched in the online biological function annotation tool PANTHER (http://www.pantherdb.org) and UNIPROT knowledge database (http://www.uniprot.org). Most of the proteins are identified in the cytoplasm (45 %) and 23 % in nucleus and mitochondrion. Functional classification shows that 44 % (n = 11) of differentially expressed protein spots are involved in mitochondrial metabolism while 24 % (n = 6), 16 % (n = 4) and 16 % (n = 4) of protein spots are associated with cytoskeleton, metabolism and other not specified functions, respectively (Table 1).

**Fig. 4:**
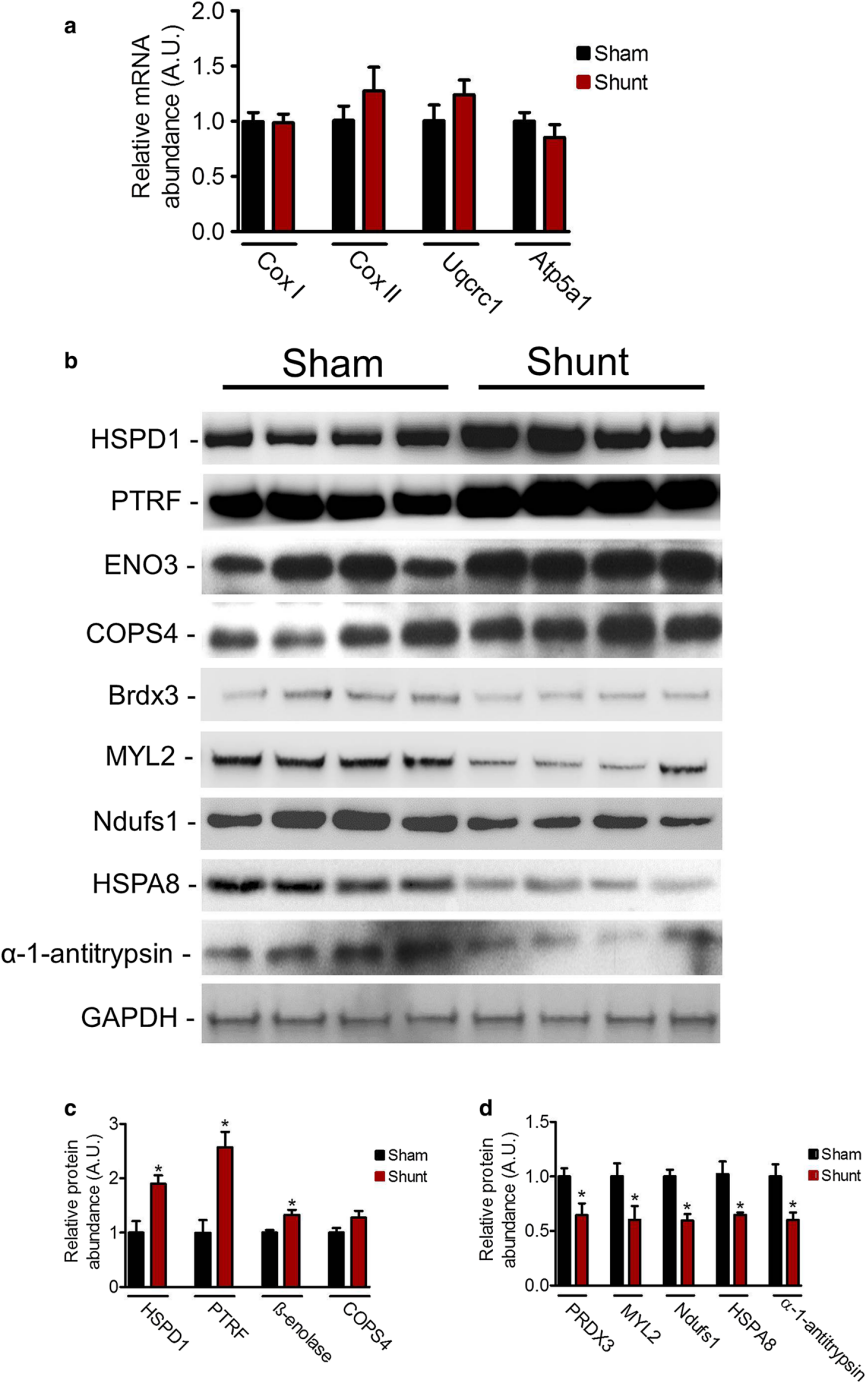
Validation of proteomics data. **a** Real time of mtDNA gene *Cox I*, *Cox II*, *Uqcrc1* and nuclear genes *Atp5a1*. **b** Representative Western blots showing change in the expression of 60 kDa heat shock protein, mitochondrial (HSPD1), Polymerase I and transcript release factor (PTRF), ß-enolase (Eno3), COP9 signalosome complex subunit 4 (COPS4), Thioredoxin-dependent peroxide reductase, mitochondrial (PRDX3), Myosin regulatory light chain 2, ventricular/cardiac muscle isoform (MYL2), NADH-ubiquinone oxidoreductase 75 kDa subunit, mitochondrial (Ndufs1), Heat shock cognate 71 kDa protein (HSPA8), and α-1-antitrypsin in respective experimental groups. **c, d** Graphs showing relative band intensity as revealed by Western blot analyses. Values are mean ± SEM; n = 4–5/group; analysis per heart in duplicate; *P < 0.05 vs. sham

We identified 7 differentially expressed electron transport chain (ETC) subunits. Of these, five ETC subunits (≈72 %), specifically four subunits of complex V and one subunit of complex III, were significantly more abundant (Table 1). To investigate the level of regulation of mitochondrial protein expression in VO, we performed real time PCR for mRNAs representing nuclear and mtDNA-encoded mitochondrial proteins. Neither mRNAs for the mtDNA gene (*Cox I, Cox II, Uqcrc1*), nor chromosomal genes (*Atp5a1*) encoding proteins were differentially expressed by VO (Fig. 4a).

### Validation by western blotting

We selected some differentially expressed proteins identified by 2-DE for immunoblotting. Consistently shunt induced-VO resulted in significant increased expression of 60 kDa heat shock protein, mitochondrial (HSPD1), Polymerase I and transcript release factor (PTRF) and ß-enolase (Fig. 4b, c), and marked decreased expression of Thioredoxin-dependent peroxide reductase, mitochondrial (PRDX3), Myosin regulatory light chain 2, ventricular/cardiac muscle isoform (MYL2), NADH-ubiquinone oxidoreductase 75 kDa subunit, mitochondrial (Ndufs1), Heat shock cognate 71 kDa protein (HSPA8) and α-1-antitrypsin (Fig. 4b, d). Signalosome complex subunit 4 (COPS4) signalosome complex subunit 4 (COPS4) showed a trend towards an increased expression in shunt mice but did not reach statistical significance (P = 0.09; Fig. 4b, c).

### Protein networks involved in short-term preload

**Fig. 5:**
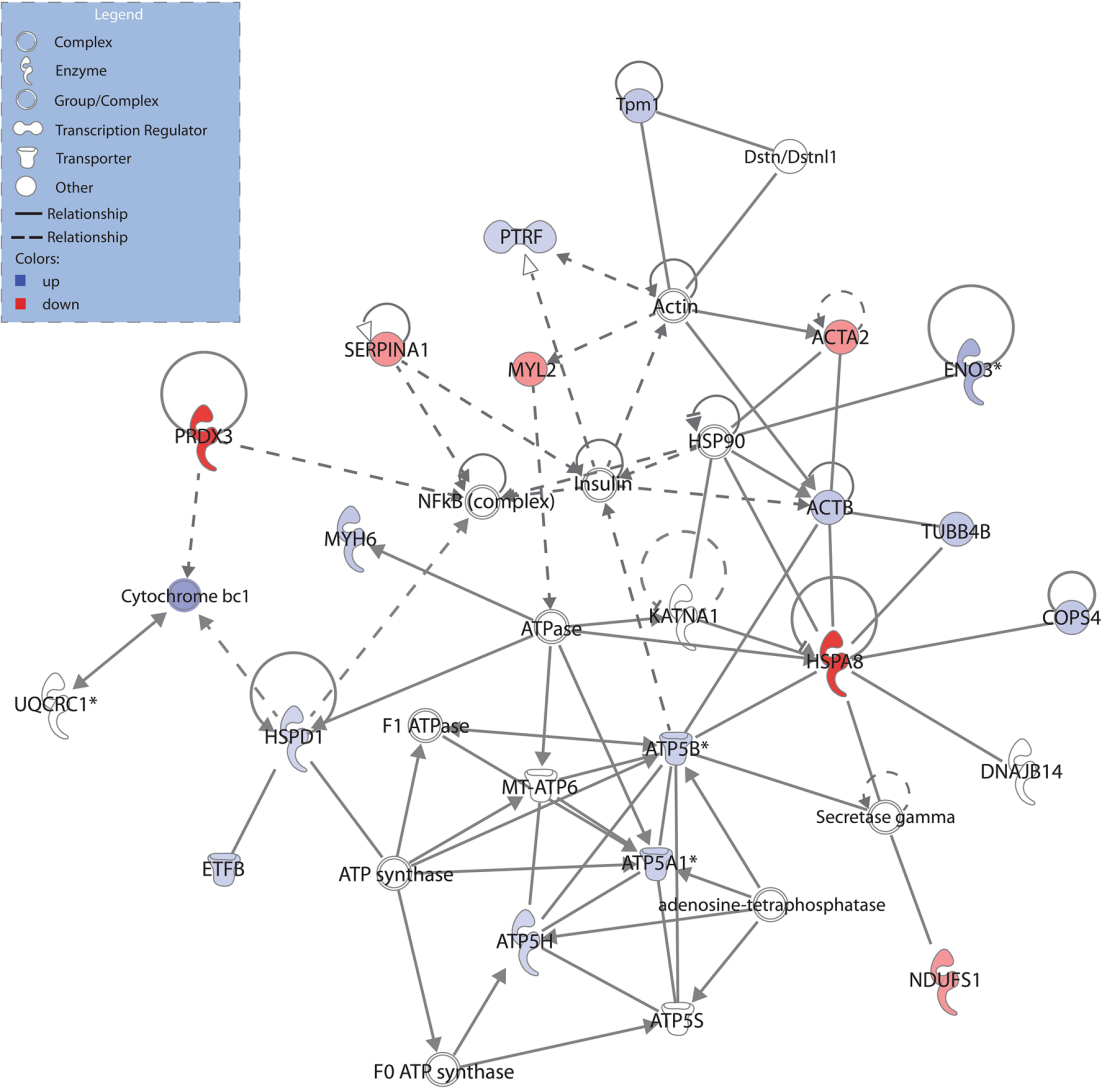
Ingenuity pathway analyses (IPA). The global differentially expressed proteins between sham and shunt were uploaded. *Colored* nodes are molecules detected from proteomic analysis, and nodes with *white* background are molecules forecasted by the IPA. *Lines* indicate interactions, with the *arrowheads* indicating directionality. Absence of arrowheads refers to a binding interaction. *Dotted line* indicates an inferred or indirect interaction

Ingenuity pathways analysis (IPA) software was selected to generate systematic network analysis of the differentially expressed proteins to elucidate integrated signaling pathways under VO. Counterparts of all these protein genes were imported to the IPA module. The generated network was attributed to the functions of energy production, nucleic acid metabolism, small molecule biochemistry, and has 35 genes (the maximum number of genes per network), among which 19 differential protein genes are involved in this network (score = 56, highly significant) (Fig. 5).

## Discussion

Proteomics provides a powerful experimental approach for observing the global changes in protein expression (level and profile) in cells, tissues or whole organs, in both physiological and pathological conditions. Several studies have examined differences in protein expression developed in different subcellular compartments of hearts and alteration in the protein expression due to afterload-induced cardiac hypertrophy [15, 16]. However, little is known about the alterations induced by preload. Thus, we have chosen a proteomic approach in a mouse model of VO-induced myocardial hypertrophy by aortocaval shunt operation and report proteomic changes after 1 week.

As predicted, VO in our murine model resulted in LV hypertrophy and LV dilatation with maintained LV function. Upon comparison between shunt and sham controls, we obtained 25 differentially displayed spots, corresponding to 21 unique proteins. It has to be highlighted that the number of spots is different from the number of the identified unique proteins, as multiple spots are generated by the same protein likely representing post-translational modifications of the protein. We found preload-induced expression changes of the following functional protein clusters: (1) myofilaments and intermediate filaments, (2) energy metabolism, and (3) other interesting proteins like α-1-antitrypsin and PTRF.

### Myofilaments and intermediate filaments

In agreement with previous studies from other cardiac hypertrophy models [17], we found an up-regulation of contractile proteins like actin and myosin VI. We also found a down-regulation of the myosin regulatory light chain 2 (MYL2). MYL2 is a cardiac-specific protein that dimerizes with cardiac myosin beta heavy chain, and its phosphorylation by Ca^2+^ triggers cardiac contraction. MYL2 mutations are associated with heart failure and familial hypertrophic cardiomyopathy [18, 19]. Li et al. [20] found a correlation between decreased MYL2 protein level and the stage of heart failure, but the analyzed samples were from the right atrium and therefore a regulation in the ventricle has not been described before. But the reduced expression of the measured spot could also be due to reduced phosphorylation. Decreased phosphorylation of MYL2 is associated with hypertrophy and fibrosis [21]. Therefore a change in MYL2 expression or phosphorylation might contribute to hypertrophy and over a longer time to heart failure development.

### Energy metabolism

Also not surprisingly, proteins of the energy metabolism were differentially expressed after VO induction. We found an up-regulation of ß-enolase protein, which mediates the penultimate step of glycolysis, converting 2-phosphoglycerate to phosphoenolpyruvate, and plays an important role in glycolytic energy production. Increased expression is likely to cope with the increased metabolic demands. Lactate dehydrogenase was significantly down-regulated, implying a shift to oxidative metabolism. Consistently, we identified five abundantly expressed ETC subunits, suggesting enhanced oxidative mitochondrial machinery in VO. Interestingly gene expression levels of mitochondrial Complex 1 (*Cox I* and *Cox II*), Complex III (*Uqcrc1*) and Complex V (*Atp5a1*) were not differentially expressed after shunt suggesting that the VO affects the expression of mitochondrial proteins at a post-transcriptional level. Previously it was reported that post-translational modification largely affects the mitochondrial protein levels [22–24].

Unpublished results from our group show that in pressure overload the regulation of mitochondrial proteins is nearly absent after 1 week. Moreover, several reports demonstrated a substantial defect in cardiac oxidative capacity and ETC subunits in pressure overload-induced heart failure [25, 26]. Because it is known that energy deficiency may contribute to heart failure [27, 28], enhancing respiratory chain machinery may prove to be beneficial in heart failure as an attempt to use an ‘oxygen-efficient’ energy substrate for ATP synthesis [29]. Moreover, the up-regulation of ATP synthase subunit alpha protein fits to our previously published in vitro results [17]. There we could show that preload leads to an increase in metabolic proteins in a model of rabbit isolated cardiac muscle strips.

### Extracellular matrix (ECM) remodeling

Although cardiomyocytes constitute 70 % of the cardiac tissue mass, they represent only about 30 % of the cell population in the heart. The other predominant cell type is the fibroblast [30]. Fibroblasts actively synthesize and secrete ECM and contribute to the propagation of the electric signals that orchestrate cardiac contraction [31]. The remodeling of ECM proteins seems to be one of the important mechanisms for the early adaptive dilatation of the heart after induction of VO, but persistent proteolytic activity might also be a mechanism for the progression to heart failure [32]. Alpha-1-antitrypsin, a member of the serine protease inhibitor family with anti-inflammatory and immunomodulatory properties [33], was recently reported to have a cytoprotective role in the endothelial cells exposed to ischemia reperfusion injury [34]. Here we found that α-1-antitrypsin was significantly down-regulated. This would lead to a higher elastin degradation rate in the heart. These results fit to studies from Zheng et al. [32] and Stewart et al. [35] showing an increased degradation of the ECM after induction of VO.

### Polymerase I and transcript release factor protein

Furthermore, we demonstrated that PTRF was up-regulated in VO. PTRF protein is involved in the transcription release of pre-ribosomal RNA and polymerase I from the DNA and regulates rRNA transcription by promoting the dissociation of transcription complexes and the reinitiation of polymerase I [36]. PTRF is localized in the caveolae of various cell types, including myocytes, endothelial cells and fibroblasts [37, 38], and that insulin treatment can induce its nuclear translocation [39]. The increase in the protein expression of PRTF in the VO model might be related to an increase in cell length [5]. Or it can be speculated that the number of caveolae might be changed in VO hypertrophy. Mutations in the PTRF gene lead to fatal cardiac arrhythmia and long-QT-syndrome [40], indicating a relevant role in the heart.

### Analysis of the preload interaction network

Cardiovascular diseases are complicated diseases that are controlled by complex regulation network. The customized pathways disclosed by IPA have important implications for the understanding of the pathogenesis of favorable remodeling induced by preload. It is noteworthy that most of the proteins identified are interconnected on the same network that primarily involves structural and metabolic proteins and enzymes involved in energy metabolism and nucleic acid metabolism.

### Potential limitation

The proteome we map is a mixture of proteins not only from cardiomyocytes but also from other cell types present in the heart, including fibroblasts, endothelial cells, and smooth muscle cells. Because the heart functions as an organ, and not as isolated cells, it can be viewed as an advantage to capture the proteome dynamics of the whole heart, rather than restricted only to the cardiomyocytes. Our rationale for examining the entire heart, then, was that we wanted to map the proteomic changes in a state as close as possible to that in vivo, which would be impossible upon cardiomyocytes isolation by enzymatic dissociation.

The nature of proteomic technique makes certain potentially relevant proteins difficult to interrogate, and their absence among the proteins identified in our study does not exclude a potential role in preload-induced eccentric remodeling. Ion channel proteins show relatively low-level expression compared to structural, contractile and metabolic proteins, explaining the lack of ion channel subunits among the differentially expressed proteins identified.

In the current study we have not studied the diastolic function, but previously we and others reported an impaired diastolic function in the mouse model upon exposure to VO owing to increased titin stiffness [41, 42].

## Conclusions

In the present study we concluded that the preload-induced eccentric cardiac hypertrophy leads to an adaptive change in the proteome with up-regulation of some mitochondrial and contractile proteins. Furthermore down-regulation of α-1-antitrypsin might contribute to the ECM remodeling leading to early dilatation. Further exploration of these proteomic data will contribute to understanding the pathogenesis of VO induced-myocardial remodeling. Besides some of the proteins found to be differentially abundant may be candidate biomarkers for diagnosis and prognosis of VO-induced myocardial remodeling.

## Additional file

10.1186/s12967-016-0898-5 MS/MS analysis table for identified proteins.
