# Supplementary material for: Proteomic analysis of short-term preload-induced eccentric cardiac hypertrophy
Source: J Transl Med. 2016 May 27;14:149. doi: 10.1186/s12967-016-0898-5 (PMC4884361; doi:10.1186/s12967-016-0898-5)
Supplement: Supplementary file 1 — 10.1186/s12967-016-0898-5 MS/MS analysis table for identified proteins. [file 12967_2016_898_MOESM1_ESM.doc]

**Additional file 1: Table S1**

MS/MS analysis table for identified proteins.

| Spot no. | Accession no. | Mass^1^ (kDa) | pI^4^ | Mascot  Score^2^ | Peptide matched^3^ | Protein name | MS/MS Analysis |
| --- | --- | --- | --- | --- | --- | --- | --- |
| 1 | P22599 | 45.9 | 5.32 | 64 | 3 | α-1-antitrypsin 1-2 | **1** MTPSISWGLL LLAGLCCMVP SFLAEDVQET DTSQK**DQSPA SHEIATNLGD**  **51 FAISLYR**ELV HQSNTSNIFF SPVSIATAFA MLSLGSKGDT HTQILEGLQF  **101** NLTQTSEADI HKSFQHLLQT LNRPDSELQL STGNGLFVNN DLKLVEKFLE  **151** EAKNHYQAEV FSVNFAESEE AKKVINDFVE KGTQGKIVEA VKELDQDTVF  **201** ALANYILFKG KWKKPFDPEN TEEAEFHVDK STTVKVPMMM LSGMLDVHHC  **251** SILSSWVLLM DYAGNASAVF LLPEDGKMQH LEQTLNKELI SKILLNRRRR  **301** LVQIHIPR**LS ISGDYNLKTL MSPLGITR**IF NNGADLSGIT EENAPLKLSK  **351** AVHKAVLTID ETGTEAAAAT VFEAVPMSMP PILRFDHPFL FIIFEEHTQS  **401** PIFVGKVVDP THK  **Start-End Observed Mr(expt) Mr(calc) Delta Miss Sequence**  **36 - 57 802.4268 2404.2586 2404.1659 0.0927 0 K.DQSPASHEIATNLGDFAISLYR.E**  **309- 318 555.3065 1108.5984 1108.5764 0.0220 0 R.LSISGDYNLK.T**  **319 - 328 552.8185 1103.6224 1103.6009 0.0216 0 K.TLMSPLGITR.I** |
| 3 | P21550 | 46.9 | 6.73 | 195 | 6 | β- enolase | **1** MAMQKIFARE ILDSRGNPTV EVDLHTAKGR FRAAVPSGAS TGIYEALELR  **51** DGDKARYLGK GVLKAVEHIN KTLGPALLEK KLSVVDQEKV DKFMIELDGT  **101** ENKSKFGANA ILGVSLAVCK AGAAEKGVPL YRHIADLAGN PDLVLPVPAF  **151** NVINGGSHAG NKLAMQEFMI LPVGASSFKE AMRIGAEVYH HLKGVIKAKY  **201** GKDATNVGDE GGFAPNILEN NEALELLK**TA IQAAGYPDK**V VIGMDVAASE  **251** FYRNGKYDLD FKSPDDPARH ISGEKLGELY KNFIQNYPVV SIEDPFDQDD  **301** WATWTSFLSG VDIQIVGDDL TVTNPKRIAQ AVEKKACNCL LLK**VNQIGSV**  **351 TESIQACK**LA QSNGWGVMVS HRSGETEDTF IADLVVGLCT GQIKTGAPCR  **401** SERLAK**YNQL MRIEEALGDK AVFAGR**KFRN PKAK    **Start-End Observed Mr(expt) Mr(calc) Delta Miss Sequence**  **229 - 239 567.8341 1133.6536 1133.5717 0.0820 0 K.TAIQAAGYPDK.V**  **344 - 358 817.4811 1632.9476 1632.8141 0.1335 0 K.VNQIGSVTESIQACK.L**  **407 - 412 412.7361 823.4576 823.4010 0.0566 0 K.YNQLMR.I**  **407 - 412 420.7343 839.4540 839.3959 0.0581 0 K.YNQLMR.I**  **413 - 420 437.7576 873.5006 873.4443 0.0563 0 R.IEEALGDK.A**  **413 - 426 492.6300 1474.8682 1474.7780 0.0902 1 R.IEEALGDKAVFAGR.K** |
| 4 | P68372 | 49.7 | 4.79 | 93 | 3 | Tubulin beta-2C chain | **1** MREIVHLQAG QCGNQIGAKF WEVISDEHGI DPTGTYHGDS DLQLERINVY  **51** YNEATGGKYV PRAVLVDLEP GTMDSVRSGP FGQIFRPDNF VFGQSGAGNN  **101** WAKGHYTEGA ELVDSVLDVV RKEAESCDCL QGFQLTHSLG GGTGSGMGTL  **151** LISKIREEYP DR**IMNTFSVV PSPK**VSDTVV EPYNATLSVH QLVENTDETY  **201** CIDNEALYDI CFRTLKLTTP TYGDLNHLVS ATMSGVTTCL R**FPGQLNADL**  **251 R**KLAVNMVPF PRLHFFMPGF APLTSRGSQQ YRALTVPELT QQMFDAKNMM  **301** AACDPRHGRY LTVAAVFRGR MSMKEVDEQM LNVQNKNSSY FVEWIPNNVK  **351** **TAVCDIPPR**G LKMSATFIGN STAIQELFKR ISEQFTAMFR RKAFLHWYTG  **401** EGMDEMEFTE AESNMNDLVS EYQQYQDATA EEEGEFEEEA EEEVA    **Start-End Observed Mr(expt) Mr(calc) Delta Miss Sequence**  **163 - 174 668.3842 1334.7538 1334.6904 0.0634 0 R.IMNTFSVVPSPK.V**  **242 - 251 565.8262 1129.6378 1129.5880 0.0499 0 R.FPGQLNADLR.K**  **351 - 359 514.7882 1027.5618 1027.5121 0.0498 0 K.TAVCDIPPR.G** |
| 5 | P62737 | 41.9 | 5.23 | 128 | 4 | Actin, aortic smooth muscle | **1** MCEEEDSTAL VCDNGSGLCK **AGFAGDDAPR** AVFPSIVGRP RHQGVMVGMG  **51** QKDSYVGDEA QSKRGILTLK YPIEHGIITN WDDMEKIWHH SFYNELRVAP  **101** EEHPTLLTEA PLNPKANREK MTQIMFETFN VPAMYVAIQA VLSLYASGRT  **151** TGIVLDSGDG VTHNVPIYEG YALPHAIMRL DLAGRDLTDY LMKILTER**GY**  **201 SFVTTAER**EI VRDIKEKLCY VALDFENEMA TAASSSSLEK SYELPDGQVI  **251** TIGNERFRCP ETLFQPSFIG MESAGIHETT YNSIMKCDID IRKDLYANNV  **301** LSGGTTMYPG IADRMQK**EIT ALAPSTMK**IK IIAPPERKYS VWIGGSILAS  **351** LSTFQQMWIS KQEYDEAGPS IVHRKCF    **Start-End Observed Mr(expt) Mr(calc) Delta Miss Sequence**  **21 - 30 488.7531 975.4916 975.4410 0.0506 0 K.AGFAGDDAPR.A**  **199 - 208 565.7990 1129.5834 1129.5404 0.0431 0 R.GYSFVTTAER.E**  **318 - 328 581.3429 1160.6712 1160.6111 0.0602 0 K.EITALAPSTMK.I** |
| 7 | Q9CZ13 | 52.7 | 5.75 | 554 | 24 | Cytochrome b-c1 complex subunit 1 | **1** MAASAVCRAA CSGTQVLLRT RRSPALLRLP ALRGTATFAQ ALQSVPETQV  **51** SILDNGLRVA SEQSSHATCT VGVWIDAGSR YETEK**NNGAG YFLEHLAFK**G  **101** TK**NRPGNALE KEVESIGAHL NAYSTREHTA YLIK**ALSKDL PK**VVELLADI**  **151 VQNSSLEDSQ IEK**ERDVILR EMQENDASMQ NVVFDYLHAT AFQGTPLAQA  **201** VEGPSENVRR LSR**TDLTDYL NR**NYKAPRMV LAAAGGVEHQ QLLDLAQKHL  **251** SSVSR**VYEED AVPGLTPCRF TGSEIR**HRDD ALPLAHVAIA VEGPGWANPD  **301** NVTLQVANAI IGHYDCTCGG GVHLSSPLAS VAVANKLCQS FQTFNISYSD  **351** TGLLGAHFVC DAMSIDDMVF FLQGQWMRLC TSATESEVTR GKNILR**NALV**  **401 SHLDGTTPVC EDIGRSLLTY GRRIPLAEWE SRIQEVDAQM LR**DICSKYFY  **451** DQCPAVAGYG PIEQLPDYNR IR**SGMFWLR**F  **Start-End Observed Mr(expt) Mr(calc) Delta Miss Sequence**  **86 - 99 790.9559 1579.8972 1579.7783 0.1189 0 K.NNGAGYFLEHLAFK.G**  **103 - 111 499.7839 997.5532 997.5305 0.0228 0 K.NRPGNALEK.E**  **112 - 126 549.6475 1645.9207 1645.8060 0.1147 0 K.EVESIGAHLNAYSTR.E**  **127 - 134 487.7831 973.5516 973.5233 0.0284 0 R.EHTAYLIK.A**  **143 - 163 1165.1959 2328.3772 2328.2060 0.1712 0 K.VVELLADIVQNSSLEDSQIEK.E**  **214 - 222 555.8075 1109.6004 1109.5353 0.0652 0 R.TDLTDYLNR.N**  **256 - 269 803.4369 1604.8592 1604.7505 0.1088 0 R.VYEEDAVPGLTPCR.F**  **270 - 276 405.2251 808.4356 808.4079 0.0277 0 R.FTGSEIR.H**  **397 - 415 685.3870 2053.1392 2052.9899 0.1493 0 R.NALVSHLDGTTPVCEDIGR.S**  **416 - 422 405.2486 808.4826 808.4443 0.0384 0 R.SLLTYGR.R**  **423 - 432 628.8789 1255.7432 1255.6673 0.0760 1 R.RIPLAEWESR.I**  **433 - 442 601.8484 1201.6822 1201.6125 0.0698 0 R.IQEVDAQMLR.D**  **473 - 479 448.7560 895.4974 895.4374 0.0600 0 R.SGMFWLR.F** |
| 8 | Q9DCW4 | 27.6 | 8.24 | 79 | 3 | Electron transfer flavoprotein subunit beta | **1** MAELRALVAV KRVIDFAVKI RVKPDKSGVV TDGVKHSMNP FCEIAVEEAV  **51** RLKEKKLVKE IIAVSCGPSQ CQETIRTALA MGADRGIHVE IPGAQAESLG  **101** PLQVARVLAK LAEKEKVDLL FLGKQAIDDD CNQTGQMTAG LLDWPQGTFA  **151** SQVTLEGDKV KVER**EIDGGL ETLR**LKLPAV VTADLRLNEP RYATLPNIMK  **201** AKKKKIEVVK **AGDLGVDLTS KVSVISVEEP PQR**SAGVKVE TTEDLVAKLK  **251** EVGRI    **Start-End Observed Mr(expt) Mr(calc) Delta Miss Sequence**  **165 - 174 551.8320 1101.6494 1101.5666 0.0829 0 R.EIDGGLETLR.L**  **211 - 221 538.3221 1074.6296 1074.5557 0.0739 0 K.AGDLGVDLTSK.V**  **222 - 233 670.4125 1338.8104 1338.7143 0.0961 0 K.VSVISVEEPPQR.S** |
| 11 | Q3U9G0 | 70.8 | 5.37 | 53 | 3 | Heat shock cognate 71 kDa protein | **1** MSKGPAVGID LGTTYSCVGV FQHGK**VEIIA NDQGNRTTPS YVAFTDTER**L  **51** IGDAAKNQVA MNPTNTVFDA KRLIGRRFDD AVVQSDMKHW PFMVVNDAGR  **101** PKVQVEYKGE TKSFYPEEVS SMVLTKMKEI AEAYLGKTVT NAVVTVPAYF  **151** NDSQRQATKD AGTIAGLNVL RIINEPTAAA IAYGLDKKVG AERNVLIFDL  **201** GGGTFDVSIL TIEDGIFEVK STAGDTHLGG EDFDNRMVNH FIAEFKRKHK  **251** KDISENKRAV RRLRTACERA KRTLSSSTQA SIEIDSLYEG IDFYTSITRA  **301** RFEELNADLF R**GTLDPVEK**A LRDAKLDKSQ IHDIVLVGGS TRIPKIQKLL  **351** QDFFNGKELN KSINPDEAVA YGAAVQAAIL SGDKSENVQD LLLLDVTPLS  **401** LGIETAGGVM TVLIKRNTTI PTKQTQTFTT YSDNQPGVLI QVYEGERAMT  **451** KDNNLLGKFE LTGIPPAPRG VPQIEVTFDI DANGILNVSA VDKSTGKENK  **501** ITITNDKGRL SKEDIERMVQ EAEKYKAEDE KQRDKVSSKN SLESYAFNMK  **551** ATVEDEKLQG KINDEDKQKI LDKCNEIISW LDKNQTAEKE EFEHQQKELE  **601** KVCNPIITKL YQSAGGMPGG MPGGFPGGGA PPSGGASSGP TIEEVD    **Start-End Observed Mr(expt) Mr(calc) Delta Miss Sequence**  **26 - 36 614.8599 1227.7052 1227.6207 0.0845 0 K.VEIIANDQGNR.T**  **37 - 49 744.4073 1486.8000 1486.6940 0.1060 0 R.TTPSYVAFTDTER.L**  **312 - 319 429.7641 857.5136 857.4494 0.0642 0 R.GTLDPVEK.A** |
| 12 | Q3U774 | 56.2 | 5.19 | 91 | 3 | ATP synthase subunit beta, mitochondrial | **1** MLSLVGRVAS ASASGALRGL SPSAALPQAQ LLLRAAPAGV HPARDYAAQA  **51** SAAPKAGTAT GRIVAVIGAV VDVQFDEGLP PILNALEVQG RDSRLVLEVA  **101** QHLGESTVRT IAMDGTEGLV RGQKVLDSGA PIKIPVGPET LGRIMNVIGE  **151** PIDERGPIKT KQFAPIHAEA PEFIEMSVEQ EILVTGIKVV DLLAPYAKGG  **201** KIGLFGGAGV GKTVLIMELI NNVAKAHGGY SVFAGVGERT REGNDLYHEM  **251** IESGVINLKD ATSK**VALVYG QMNEPPGAR**A RVALTGLTVA EYFRDQEGQD  **301** VLLFIDNIFR FTQAGSEVSA LLGRIPSAVG YQPTLATDMG TMQERITTTK  **351** KGSITSVQAI YVPADDLTDP APATTFAHLD ATTVLSRAIA ELGIYPAVDP  **401** LDSTSR**IMDP NIVGNEHYDV AR**GVQKILQD YKSLQDIIAI LGMDELSEED  **451** KLTVSRARKI QRFLSQPFQV AEVFTGHMGK LVPLKETIKG FQQILAGEYD  **501** HLPEQAFYMV GPIEEAVAKA DKLAEEHGS    **Start-End Observed Mr(expt) Mr(calc) Delta Miss Sequence**  **265 - 279 809.4380 1616.8614 1616.7981 0.0634 0 K.VALVYGQMNEPPGAR.A**  **407 - 422 614.9872 1841.9398 1841.8730 0.0668 0 R.IMDPNIVGNEHYDVAR.G** |
| 13 | P16125 | 36.5 | 5.70 | 94 | 4 | L-lactate dehydrogenase B chain | **1** MATLKEKLIA SVADDEAAVP NNKITVVGVG QVGMACAISI LGKSLADELA  **51** LVDVLEDKLK GEMMDLQHGS LFLQTPK**IVA DKDYSVTANS K**IVVVTAGVR  **101** QQEGESRLNL VQRNVNVFKF IIPQIVKYSP DCTIIVVSNP VDILTYVTWK  **151** LSGLPKHRVI GSGCNLDSAR FRYLMAEKLG IHPSSCHGWI LGEHGDSSVA  **201** VWSGVNVAGV SLQELNPEMG TDNDSENWKE VHKMVVDSAY EVIKLKGYTN  **251** WAIGLSVADL IESMLKNLSR IHPVSTMVKG MYGIENEVFL SLPCILNAR**G**  **301 LTSVINQK**LK DDEVAQLRKS ADTLWDIQKD LKDL  **Start-End Observed Mr(expt) Mr(calc) Delta Miss Sequence**  **78 - 91 504.2672 1509.7798 1509.7675 0.0123 1 K.IVADKDYSVTANSK.I**  **83 - 91 492.7451 983.4756 983.4560 0.0197 0 K.DYSVTANSK.I**  **300 - 308 480.2776 958.5406 958.5447 -0.0041 0 R.GLTSVINQK.L** |
| 14 | P51667 | 18.8 | 4.86 | 229 | 7 | Myosin regulatory light chain 2, ventricular/cardiac muscle isoform | **1** MAPKKAKKRI EGGSSNVFSM FEQTQIQEFK **EAFTIMDQNR DGFIDKNDLR**  **51 DTFAALGRVN VKNEEIDEMI K**EAPGPINFT VFLTMFGEKL KGADPEETIL  **101** NAFKVFDPEG KGSLKADYVR EMLTTQAERF SKEEIDQMFA AFPPDVTGNL  **151** DYKNLVHIIT HGEEKD  **Start-End Observed Mr(expt) Mr(calc) Delta Miss Sequence**  **31 - 40 612.7905 1223.5664 1223.5605 0.0060 0 K.EAFTIMDQNR.D**  **41 - 50 596.7942 1191.5738 1191.5884 -0.0145 1 R.DGFIDKNDLR.D**  **41 - 50 596.7997 1191.5848 1191.5884 -0.0035 1 R.DGFIDKNDLR.D**  **51 - 58 425.7216 849.4286 849.4345 -0.0058 0 R.DTFAALGR.V**  **59 - 71 520.9181 1559.7325 1559.7865 -0.0540 1 R.VNVKNEEIDEMIK.E** |
| 16 | Q91VD9 | 79.6 | 5.51 | 475 | 16 | NADH-ubiquinone oxidoreductase 75 kDa subunit, mitochondrial | **1** MLRIPIKRAL IGLSNSPKGY VRTTGTAASN LIEVFVDGQS VMVEPGTTVL  **51** QACEK**VGMQI PR**FCYHER**LS VAGNCRMCLV EIEK**APKVVA ACAMPVMKGW  **101** NILTNSEKSK KAREGVMEFL LANHPLDCPI CDQGGECDLQ DQSMMFGSDR  **151** SRFLEGKRAV EDKNIGPLVK TIMTRCIQCT RCIR**FASEIA GVDDLGTTGR**  **201 GNDMQVGTYI EK**MFMSELSG NVIDICPVGA LTSKPYAFTA RPWETRKTES  **251** IDVMDAVGSN IVVSTRTGEV MRILPRMHED INEEWISDKT RFAYDGLKRQ  **301** R**LTEPMVR**NE KGLLTYTSWE DALSRVAGML QNFEGNAVAA IAGGLVDAEA  **351** LVALKDLLNK VDSDNLCTEE IFPTEGAGTD LRSNYLLNTT IAGVEEADVV  **401** LLVGTNPR**FE APLFNAR**IRK SWLHNDLKVA LIGSPVDLTY R**YDHLGDSPK**  **451 ILQDIASGR**H SFCEVLKDAK KPMVVLGSSA LQR**DDGAAIL AAVSNMVQK**I  **501** RVTTGVAAEW KVMNILHRIA SQVAALDLGY KPGVEAIRKN PPK**MLFLLGA**  **551 DGGCITR**QDL PKDCFIVYQG HHGDVGAPMA DVILPGAAYT EK**SATYVNTE**  **601 GR**AQQTK**VAV TPPGLAR**EDW KIIRALSEIA GITLPYDTLD QVRNRLEEVS  **651** PNLVRYDDIE ETNYFQQASE LAKLVNQEVL ADPLVPPQLT IK**DFYMTDSI**  **701 SR**ASQTMAKC VKAVTEGAQA VEEPSIC    **Start-End Observed Mr(expt) Mr(calc) Delta Miss Sequence**  **56 - 62 408.7151 815.4156 815.4324 -0.0167 0 K.VGMQIPR.F**  **69 - 76 438.7082 875.4018 875.4283 -0.0265 0 R.LSVAGNCR.M**  **77 - 84 511.2437 1020.4728 1020.4984 -0.0255 0 R.MCLVEIEK.A**  **185 - 200 804.8806 1607.7466 1607.7791 -0.0325 0 R.FASEIAGVDDLGTTGR.G**  **201 - 212 677.8120 1353.6094 1353.6235 -0.0140 0 R.GNDMQVGTYIEK.M**  **302 - 308 423.2209 844.4272 844.4477 -0.0204 0 R.LTEPMVR.N**  **409 - 417 532.7487 1063.4828 1063.5451 -0.0622 0 R.FEAPLFNAR.I**  **442 - 450 516.2350 1030.4554 1030.4720 -0.0165 0 R.YDHLGDSPK.I**  **451 - 459 486.7600 971.5054 971.5400 -0.0345 0 K.ILQDIASGR.H**  **484 - 499 801.9109 1601.8072 1601.8083 -0.0010 0 R.DDGAAILAAVSNMVQK.I**  **544 - 557 762.3896 1522.7646 1522.7636 0.0010 0 K.MLFLLGADGGCITR.Q**  **593 - 602 549.2476 1096.4806 1096.5149 -0.0342 0 K.SATYVNTEGR.A**  **608 - 617 490.7817 979.5488 979.5815 -0.0326 0 K.VAVTPPGLAR.E**  **693 - 702 625.7646 1249.5146 1249.5285 -0.0139 0 K.DFYMTDSISR.A** |
| 18 | O88544 | 46.2 | 5.57 | 237 | 9 | COP9 signalosome complex subunit 4 | **1** MAAAVRQDLA QLMNSSGSHK DLAGKYRQIL EKAIQLSGTE QLEALKAFVE  **51** AMVNENVSLV ISRQLLTDFC THLPNLPDST AK**EVYHFTLE K**IQPR**VISFE**  **101 EQVASIR**QHL ASIYEKEEDW RNAAQVLVGI PLETGQKQYN VDYKLETYLK  **151** IARLYLEDDD PVQAEAYINR ASLLQNESTN EQLQIHYKVC YARVLDYRR**K**  **201 FIEAAQRYNE LSYKTIVHES ER**LEALKHAL HCTILASAGQ QRSRMLATLF  **251** KDERCQQLAA YGILEKMYLD RIIRGNQLQE FAAMLMPHQK **ATTADGSSIL**  **301 DRAVIEHNLL SASK**LYNNIT FEELGALLEI PAAKAEK**IAS QMITEGR**MNG  **351** FIDQIDGIVH FETR**EALPTW DK**QIQSLCFQ VNNLLEKISQ TAPEWTAQAM  **401** EAQMAQ    **Start-End Observed Mr(expt) Mr(calc) Delta Miss Sequence**  **83 - 91 583.2913 1164.5680 1164.5815 -0.0135 0 K.EVYHFTLEK.I**  **96 - 107 689.3585 1376.7024 1376.7300 -0.0275 0 R.VISFEEQVASIR.Q**  **200 - 207 481.7659 961.5172 961.5345 -0.0172 1 R.KFIEAAQR.Y**  **208 - 214 458.7227 915.4308 915.4338 -0.0029 0 R.YNELSYK.T**  **215 - 222 485.7535 969.4924 969.4879 0.0045 0 K.TIVHESER.L**  **291 - 302 603.7974 1205.5802 1205.5888 -0.0085 0 K.ATTADGSSILDR.A**  **303 - 314 641.3541 1280.6936 1280.7088 -0.0152 0 R.AVIEHNLLSASK.L**  **338 - 347 561.2789 1120.5432 1120.5546 -0.0114 0 K.IASQMITEGR.M**  )  **365 - 372 480.2386 958.4626 958.4760 -0.0133 0 R.EALPTWDK.Q** |
| 20 | Q02566 | 22.3 | 5.57 | 60 | 4 | Myosin- 6 | **1201** SVAELGEQID NLQRVKQKLE KEKSEFKLEL DDVTSNMEQI IKAKANLEKV  **1251** SRTLEDQANE YR**VKLEEAQR** SLNDFTTQRA K**LQTENGELA R**QLEEKEALI  **1301** SQLTRGKLSY TQQMEDLKRQ LEEEGKAKNA LAHALQSSRH DCDLLREQYE  **1351** EEMEAKAELQ RVLSKANSEV AQWR**TKYETD AIQR**TEELEE AKKKLAQRLQ  **1401** DAEEAVEAVN AKCSSLEKTK HRLQNEIEDL MVDVER**SNAA AAALDKK**QRN  **1451** FDKILAEWKQ KYEESQSELE SSQKEARSLS TELFKLKNAY EESLEHLETF  **1501** KRENKNLQEE ISDLTEQLGE GGKNVHELEK IRKQLEVEKL ELQSALEEAE  **1551** ASLEHEEGKI LRAQLEFNQI KAEIERKLAE KDEEMEQAKR NHLRMVDSLQ  **1601** TSLDAETRSR NEALRVKKKM EGDLNEMEIQ LSQANRIASE AQKHLKNSQA  **1651** HLKDTQLQLD DAVHANDDLK ENIAIVERRN NLLQAELEEL RAVVEQTERS  **1701** RKLAEQELIE TSERVQLLHS QNTSLINQKK KMESDLTQLQ TEVEEAVQEC  **1751** RNAEEKAKKA ITDAAMMAEE LKKEQDTSAH LERMKKNMEQ TIKDLQHRLD  **1801** EAEQIALKGG KKQLQKLEAR VRELENELEA EQKRNAESVK GMRKSERRIK  **1851** ELTYQTEEDK KNLMRLQDLV DKLQLKVKAY KRQAEEAEEQ ANTNLSKFRK  **1901** VQHELDEAEE RADIAESQVN KLRAKSRDIG AKKMHDEE  **Start-End Observed Mr(expt) Mr(calc) Delta Miss Sequence**  **1263 - 1270 486.7899 971.5652 971.5400 0.0253 1 R.VKLEEAQR.S**  **1282 - 1291 565.8021 1129.5896 1129.5727 0.0169 0 K.LQTENGELAR.Q**  **1375 - 1384 612.8218 1223.6290 1223.6146 0.0145 1 R.TKYETDAIQR.T**  **1437 - 1447 530.2986 1058.5826 1058.5720 0.0107 1 R.SNAAAAALDKK.Q** |
| 21 | P58771 | 32.6 | 4.69 | 59 | 4 | Tropomyosin alpha-1 chain | **1** MDAIKKKMQM LKLDKENALD RAEQAEADKK AAEDRSKQLE DELVSLQKKL  **51** KGTEDELDKY SEALKDAQEK LELAEKKATD AEADVASLNR RIQLVEEELD  **101** RAQERLATAL QK**LEEAEKAA DESER**GMKVI ESRAQKDEEK MEIQEIQLKE  **151** AK**HIAEDADR KYEEVARKLV IIESDLER**AE ERAELSEGKC AELEEELKTV  **201** TNNLKSLEAQ AEKYSQKEDK YEEEIKVLSD KLKEAETRAE FAERSVTKLE  **251** KSIDDLEDEL YAQKLKYKAI SEELDHALND MTSI    **Start-End Observed Mr(expt) Mr(calc) Delta Miss Sequence**  **113 - 125 492.8976 1475.6710 1475.6739 -0.0029 1 K.LEEAEKAADESER.G**  **153 - 160 463.7327 925.4508 925.4253 0.0255 0 K.HIAEDADR.K**  **161 - 167 447.7387 893.4628 893.4606 0.0022 1 R.KYEEVAR.K**  **168 - 178 657.9007 1313.7868 1313.7554 0.0314 1 R.KLVIIESDLER.A** |
| 22 | Q6ZWM3 | 41.7 | 5.29 | 86 | 6 | Actin, cytoplasmic 1 | **1** MDDDIAALVV DNGSGMCK**AG FAGDDAPR**AV FPSIVGRPRH QGVMVGMGQK  **51** **DSYVGDEAQS KR**GILTLKYP IEHGIVTNWD DMEKIWHHTF YNELR**VAPEE**  **101 HPVLLTEAPL NPK**ANREKMT QIMFETFNTP AMYVAIQAVL SLYASGRTTG  **151** IVMDSGDGVT HTVPIYEGYA LPHAILRLDL AGR**DLTDYLM K**ILTER**GYSF**  **201 TTTAER**EIVR DIKEKLCYVA LDFEQEMATA ASSSSLEKSY ELPDGQVITI  **251** GNERFRCPEA LFQPSFLGME SCGIHETTFN SIMKCDVDIR KDLYANTVLS  **301** GGTTMYPGIA DRMQKEITAL APSTMKIKII APPERKYSVW IGGSILASLS  **351** TFQQMWISK**Q EYDESGPSIV HR**KCF    **Start-End Observed Mr(expt) Mr(calc) Delta Miss Sequence**  **19 - 28 488.7498 975.4850 975.4410 0.0440 0 K.AGFAGDDAPR.A**  **51 - 62 677.8580 1353.7014 1353.6161 0.0854 1 K.DSYVGDEAQSKR.G**  **96 - 113 652.0685 1953.1837 1953.0571 0.1266 0 R.VAPEEHPVLLTEAPLNPK.A**  **184 - 191 499.7726 997.5306 997.4790 0.0516 0 R.DLTDYLMK.I**  **197 - 206 566.7963 1131.5780 1131.5197 0.0584 0 R.GYSFTTTAER.E**  **360 - 372 506.2664 1515.7774 1515.6954 0.0820 0 K.QEYDESGPSIVHR.K** |
| 24 | P63038 | 60.9 | 5.91 | 211 | 8 | 60 kDa heat shock protein, mitochondrial | **1** MLRLPTVLRQ MRPVSRALAP HLTRAYAKDV KFGADARALM LQGVDLLADA  **51** VAVTMGPKGR TVIIEQSWGS PKVTKDGVTV AKSIDLKDKY KNIGAKLVQD  **101** VANNTNEEAG DGTTTATVLA RSIAKEGFEK ISKGANPVEI RRGVMLAVDA  **151** VIAELKKQSK PVTTPEEIAQ VATISANGDK DIGNIISDAM KKVGRKGVIT  **201** VKDGKTLNDE LEIIEGMKFD RGYISPYFIN TSKGQKCEFQ DAYVLLSEKK  **251** ISSVQSIVPA LEIANAHRKP LVIIAEDVDG EALSTLVLNR LK**VGLQVVAV**  **301 KAPGFGDNR**K NQLKDMAIAT GGAVFGEEGL NLNLEDVQAH DLGK**VGEVIV**  **351 TK**DDAMLLKG KGDKAHIEKR IQEITEQLDI TTSEYEKEKL NERLAKLSDG  **401** VAVLK**VGGTS DVEVNEKK**DR **VTDALNATR**A AVEEGIVLGG GCALLRCIPA  **451** LDSLKPANED QKIGIEIIKR ALK**IPAMTIA KNAGVEGSLI VEK**ILQSSSE  **501** VGYDAMLGDF VNMVEKGIID PTKVVRTALL DAAGVASLLT TAEAVVTEIP  **551** KEEKDPGMGA MGGMGGGMGG GMF    **Start-End Observed Mr(expt) Mr(calc) Delta Miss Sequence**  **293 - 301 456.8058 911.5970 911.5804 0.0166 0 K.VGLQVVAVK.A**  **302 - 309 417.2084 832.4022 832.3828 0.0195 0 K.APGFGDNR.K**  **345 - 352 422.7626 843.5106 843.5066 0.0041 0 K.VGEVIVTK.D**  **406 - 417 617.3235 1232.6324 1232.5885 0.0440 0 K.VGGTSDVEVNEK.K**  **406 - 418 454.5803 1360.7191 1360.6834 0.0356 1 K.VGGTSDVEVNEKK.D**  **421 - 429 480.7632 959.5118 959.5036 0.0082 0 R.VTDALNATR.A**  **474 - 481 430.7600 859.5054 859.4837 0.0217 0 K.IPAMTIAK.N**  **482 - 493 608.3527 1214.6908 1214.6507 0.0402 0 K.NAGVEGSLIVEK.I** |
| 25 | P10518 | 36.0 | 6.32 | 51 | 3 | Delta-aminolevulinic acid dehydratase | **1** MHHQSVLHSG YFHPLLRSWQ TAASTVSASN LIYPIFVTDV PDDVQPIASL  **51** PGVARYGVNQ LEEMLRPLVE AGLRCVLIFG VPSRVPKDEQ GSAADSEDSP  **101** TIEAVRLLRK TFPSLLVACD VCLCPYTSHG HCGLLSENGA FLAEESRQR**L**  **151 AEVALAYAK**A GCQVVAPSDM MDGRVEAIKA ALLKHGLGNR **VSVMSYSAK**F  **201** ASCFYGPFRD AAQSSPAFGD RR**CYQLPPGA R**GLALRAVAR DIQEGADMLM  **251** VKPGLPYLDM VREVKDKHPE LPLAVYQVSG EFAMLWHGAQ AGAFDLRTAV  **301** LETMTAFRRA GADIIITYFA PQLLKWLKEE  **Start-End Observed Mr(expt) Mr(calc) Delta Miss Sequence**  **150 - 159 524.8264 1047.6382 1047.5964 0.0418 0 R.LAEVALAYAK.A**  **191 - 199 494.2630 986.5114 986.4743 0.0372 0 R.VSVMSYSAK.F**  **223 - 231 531.2839 1060.5532 1060.5124 0.0409 0 R.CYQLPPGAR.G** |
| 28 | O54724 | 43.9 | 5.43 | 56 | 2 | Polymerase I and transcript release factor | **1** MEDVTLHIVE RPYSGFPDAS SEGPEPTQGE ARATEEPSGT GSDELIKSDQ  **51** VNGVLVLSLL DKIIGAVDQI QLTQAQLEER QAEMEGAVQS IQGELSKLGK  **101** AHATTSNTVS KLLEKVRKVS VNVKTVRGSL ERQAGQIK**KL EVNEAELLR**R  **151** RNFKVMIYQD EVKLPAKLSV SKSLKESEAL PEKEGDELGE GERPEDDTAA  **201** IELSSDEAVE VEEVIEESRA ERIKRSGLRR VDDFKKAFSK EKMEKTKVRT  **251** RENLEKTRLK TKENLEKTRH TLEKRMNKLG TRLVPVERRE KLKTSRDKLR  **301** KSFTPDHVVY ARSKTAVYKV PPFTFHVKKI R**EGEVEVLK**A TEMVEVGPED  **351** DEVGAERGEA TDLLRGSSPD VHTLLEITEE SDAVLVDKSD SD    **Start-End Observed Mr(expt) Mr(calc) Delta Miss Sequence**  **139 - 149 657.4152 1312.8158 1312.7350 0.0808 1 K.KLEVNEAELLR.R**  **332 - 339 451.7686 901.5226 901.4756 0.0470 0 R.EGEVEVLK.A** |
| 30 | Q03265 | 59.7 | 9.22 | 383 | 28 | ATP synthase subunit alpha, mitochondrial | **1** MLSVRVAAAV ARALPRRAGL VSKNALGSSF VGARNLHASN TRLQKTGTAE  **51** MSSILEERIL GADTSVDLEE TGR**VLSIGDG IAR**VHGLRNV QAEEMVEFSS  **101** GLKGMSLNLE PDNVGVVVFG NDKLIKEGDV VKR**TGAIVDV PVGEELLGRV**  **151 VDALGNAIDG K**GPIGSKTRR RVGLKAPGII PR**ISVREPMQ TGIKAVDSLV**  **201 PIGR**GQR**ELI IGDR**QTGKTS IAIDTIINQK **RFNDGTDEK**K KLYCIYVAIG  **251** QKRSTVAQLV KRLTDADAMK YTIVVSATAS DAAPLQYLAP YSGCSMGEYF  **301** RDNGKHALII YDDLSKQAVA YRQMSLLLRR PPGREAYPGD VFYLHSRLLE  **351** RAAKMNDSFG GGSLTALPVI ETQAGDVSAY IPTNVISITD GQIFLETELF  **401** YKGIRPAINV GLSVSRVGSA AQTRAMKQVA GTMKLELAQY REVAAFAQFG  **451** SDLDAATQQL LSRGVRLTEL LKQGQYSPMA IEEQVAVIYA GVR**GYLDKLE**  **501 PSK**ITKFENA FLSHVISQHQ SLLGNIRSDG KISEQSDAKL KEIVTNFLAG  **551** FEP    **Start-End Observed Mr(expt) Mr(calc) Delta Miss Sequence**  **74 - 83 500.7841 999.5536 999.5713 -0.0176 0 R.VLSIGDGIAR.V**  **74 - 83 500.8270 999.6394 999.5713 0.0682 0 R.VLSIGDGIAR.V**  **74 - 83 500.8279 999.6412 999.5713 0.0700 0 R.VLSIGDGIAR.V**  **74 - 83 500.8284 999.6422 999.5713 0.0710 0 R.VLSIGDGIAR.V**  **74 - 83 500.8290 999.6434 999.5713 0.0722 0 R.VLSIGDGIAR.V**  **74 - 83 500.8304 999.6462 999.5713 0.0750 0 R.VLSIGDGIAR.V**  **74 - 83 500.8307 999.6468 999.5713 0.0756 0 R.VLSIGDGIAR.V**  **134 - 149 812.9767 1623.9388 1623.8832 0.0557 0 R.TGAIVDVPVGEELLGR.V**  **150 - 161 586.3188 1170.6230 1170.6245 -0.0014 0 R.VVDALGNAIDGK.G**  **150 - 161 586.3561 1170.6976 1170.6245 0.0732 0 R.VVDALGNAIDGK.G**  **150 - 161 586.3566 1170.6986 1170.6245 0.0742 0 R.VVDALGNAIDGK.G**  **150 - 161 586.3589 1170.7032 1170.6245 0.0788 0 R.VVDALGNAIDGK.G**  **150 - 161 586.3607 1170.7068 1170.6245 0.0824 0 R.VVDALGNAIDGK.G**  **150 - 161 586.3615 1170.7084 1170.6245 0.0840 0 R.VVDALGNAIDGK.G**  **183 - 194 453.6128 1357.8166 1357.7388 0.0778 1 R.ISVREPMQTGIK.A**  **183 - 194 458.9383 1373.7931 1373.7337 0.0594 1 R.ISVREPMQTGIK.A**  **183 - 194 458.9459 1373.8159 1373.7337 0.0822 1 R.ISVREPMQTGIK.A**  **187 - 194 452.2646 902.5146 902.4531 0.0615 0 R.EPMQTGIK.A**  **187 - 194 460.2651 918.5156 918.4481 0.0676 0 R.EPMQTGIK.A**  **195 - 204 513.7921 1025.5696 1025.5869 -0.0173 0 K.AVDSLVPIGR.G**  **195 - 204 513.8351 1025.6556 1025.5869 0.0687 0 K.AVDSLVPIGR.G**  **195 - 204 513.8352 1025.6558 1025.5869 0.0689 0 K.AVDSLVPIGR.G**  **195 - 204 513.8372 1025.6598 1025.5869 0.0729 0 K.AVDSLVPIGR.G**  **195 - 204 513.8373 1025.6600 1025.5869 0.0731 0 K.AVDSLVPIGR.G**  **208 - 214 408.2383 814.4620 814.4548 0.0072 0 R.ELIIGDR.Q**  **208 - 214 408.2598 814.5050 814.4548 0.0502 0 R.ELIIGDR.Q**  **231 - 239 541.2816 1080.5486 1080.4836 0.0651 1 K.RFNDGTDEK.K**  **494 - 503 575.3504 1148.6862 1148.6077 0.0785 1 R.GYLDKLEPSK.I** |
| 31 | Q9DCX2 | 18.7 | 5.52 | 367 | 12 | ATP synthase subunit d, mitochondrial | **1** MAGRKLALKT IDWVSFVEVM PQNQKAIGNA LK**SWNETFHA R**LASLSEKPP  **51** AIDWAYYR**AN VAKPGLVDDF EKKYNALKIP VPEDKYTALV DQEEKEDVKS**  **101 CAEFVSGSQL R**IQEYEKQLE KMRNIIPFDQ MTIDDLNEIF PETKLDKKKY  **151** PYWPHQPIEN L    **Start-End Observed Mr(expt) Mr(calc) Delta Miss Sequence**  **33 - 41 574.2659 1146.5172 1146.5206 -0.0034 0 K.SWNETFHAR.L**  **59 - 72 501.5739 1501.6999 1501.7776 -0.0778 0 R.ANVAKPGLVDDFEK.K**  **59 - 72 751.8888 1501.7630 1501.7776 -0.0146 0 R.ANVAKPGLVDDFEK.K**  **59 - 72 501.6013 1501.7821 1501.7776 0.0044 0 R.ANVAKPGLVDDFEK.K**  **59 - 73 544.3018 1629.8836 1629.8726 0.0110 1 R.ANVAKPGLVDDFEKK.Y**  **59 - 73 544.3021 1629.8845 1629.8726 0.0119 1 R.ANVAKPGLVDDFEKK.Y**  **74 - 85 462.9267 1385.7583 1385.7554 0.0028 1 K.YNALKIPVPEDK.Y**  **86 - 95 598.2888 1194.5630 1194.5768 -0.0138 0 K.YTALVDQEEK.E**  **86 - 99 556.2721 1665.7945 1665.8097 -0.0153 1 K.YTALVDQEEKEDVK.S**  **86 - 99 833.9108 1665.8070 1665.8097 -0.0027 1 K.YTALVDQEEKEDVK.S**  **100 - 111 670.8159 1339.6172 1339.6190 -0.0018 0 K.SCAEFVSGSQLR.I**  **100 - 111 670.8217 1339.6288 1339.6190 0.0098 0 K.SCAEFVSGSQLR.I** |
| 32 | Q9CZ13 | 52.7 | 5.75 | 87 | 5 | Cytochrome b-c1 complex subunit 1, mitochondrial | **1** MAASAVCRAA CSGTQVLLRT RRSPALLRLP ALRGTATFAQ ALQSVPETQV  **51** SILDNGLRVA SEQSSHATCT VGVWIDAGSR YETEKNNGAG YFLEHLAFKG  **101** TKNRPGNALE KEVESIGAHL NAYSTREHTA YLIKALSKDL PKVVELLADI  **151** VQNSSLEDSQ IEKERDVILR EMQENDASMQ NVVFDYLHAT AFQGTPLAQA  **201** VEGPSENVRR LSRTDLTDYL NRNYKAPRMV LAAAGGVEHQ QLLDLAQKHL  **251** SSVSR**VYEED AVPGLTPCRF TGSEIR**HRDD ALPLAHVAIA VEGPGWANPD  **301** NVTLQVANAI IGHYDCTCGG GVHLSSPLAS VAVANKLCQS FQTFNISYSD  **351** TGLLGAHFVC DAMSIDDMVF FLQGQWMR**LC TSATESEVTR** GKNILRNALV  **401** SHLDGTTPVC EDIGR**SLLTY GR**RIPLAEWE SR**IQEVDAQM LR**DICSKYFY  **451** DQCPAVAGYG PIEQLPDYNR IRSGMFWLRF  **Start-End Observed Mr(expt) Mr(calc) Delta Miss Sequence**  **256 - 269 803.3937 1604.7728 1604.7505 0.0224 0 R.VYEEDAVPGLTPCR.F**  **270 - 276 405.2123 808.4100 808.4079 0.0021 0 R.FTGSEIR.H**  **379 - 390 677.3300 1352.6454 1352.6242 0.0213 0 R.LCTSATESEVTR.G**  **416 - 422 405.2288 808.4430 808.4443 -0.0012 0 R.SLLTYGR.R**  **433 - 442 601.8190 1201.6234 1201.6125 0.0110 0 R.IQEVDAQMLR.D** |
| 33 | Q03265 | 59.7 | 9.22 | 51 | 3 | ATP synthase subunit alpha, mitochondrial | **1** MLSVRVAAAV ARALPRRAGL VSKNALGSSF VGARNLHASN TRLQKTGTAE  **51** MSSILEERIL GADTSVDLEE TGRVLSIGDG IARVHGLRNV QAEEMVEFSS  **101** GLKGMSLNLE PDNVGVVVFG NDKLIKEGDV VKRTGAIVDV PVGEELLGR**V**  **151 VDALGNAIDG K**GPIGSKTRR RVGLKAPGII PRISVREPMQ TGIKAVDSLV  **201** PIGRGQRELI IGDRQTGKTS IAIDTIINQK RFNDGTDEKK KLYCIYVAIG  **251** QKRSTVAQLV K**RLTDADAMK** YTIVVSATAS DAAPLQYLAP YSGCSMGEYF  **301** RDNGK**HALII YDDLSK**QAVA YRQMSLLLRR PPGREAYPGD VFYLHSRLLE  **351** RAAKMNDSFG GGSLTALPVI ETQAGDVSAY IPTNVISITD GQIFLETELF  **401** YKGIRPAINV GLSVSRVGSA AQTRAMKQVA GTMKLELAQY REVAAFAQFG  **451** SDLDAATQQL LSRGVRLTEL LKQGQYSPMA IEEQVAVIYA GVRGYLDKLE  **501** PSKITKFENA FLSHVISQHQ SLLGNIRSDG KISEQSDAKL KEIVTNFLAG  **551** FEP    **Start-End Observed Mr(expt) Mr(calc) Delta Miss Sequence**  **150 - 161 586.3254 1170.6362 1170.6245 0.0118 0 R.VVDALGNAIDGK.G**  **262 - 270 510.7706 1019.5266 1019.5070 0.0197 1 K.RLTDADAMK.Y**  **306 - 316 644.3632 1286.7118 1286.6870 0.0248 0 K.HALIIYDDLSK.Q** |
| 34 | P21550 | 46.9 | 6.73 | 87 | 3 | β-enolase | **1** MAMQKIFARE ILDSR**GNPTV EVDLHTAK**GR FRAAVPSGAS TGIYEALELR  **51** DGDKARYLGK GVLKAVEHIN KTLGPALLEK KLSVVDQEKV DKFMIELDGT  **101** ENKSKFGANA ILGVSLAVCK AGAAEKGVPL YRHIADLAGN PDLVLPVPAF  **151** NVINGGSHAG NKLAMQEFMI LPVGASSFKE AMRIGAEVYH HLKGVIKAKY  **201** GKDATNVGDE GGFAPNILEN NEALELLKTA IQAAGYPDKV VIGMDVAASE  **251** FYRNGKYDLD FKSPDDPARH ISGEKLGELY KNFIQNYPVV SIEDPFDQDD  **301** WATWTSFLSG VDIQIVGDDL TVTNPKRIAQ AVEKKACNCL LLKVNQIGSV  **351** TESIQACKLA QSNGWGVMVS HRSGETEDTF IADLVVGLCT GQIK**TGAPCR**  **401 SER**LAKYNQL MR**IEEALGDK** AVFAGRKFRN PKAK  **Start-End Observed Mr(expt) Mr(calc) Delta Miss Sequence**  **16 - 28 690.9096 1379.8046 1379.7045 0.1002 0 R.GNPTVEVDLHTAK.G**  **95 - 403 517.2957 1032.5768 1032.4771 0.0998 1 K.TGAPCRSER.**  **413 - 420 437.7587 873.5028 873.4443 0.0585 0 R.IEEALGDK.A** |
| 35 | P20108 | 28.1 | 7.15 | 117 | 4 | Thioredoxin-dependent peroxide reductase, mitochondrial | **1** MAAAAGRLLW SSVARHASAI SRSISASTVL RPVASRRTCL TDILWSASAQ  **51** GKSAFSTSSS FHTPAVTQHA PYFKGTAVVN GEFKELSLDD FKGKYLVLFF  **101** YPLDFTFVCP TEIVAFSDKA NEFHDVNCEV VAVSVDSHFS HLAWINTPRK  **151** NGGLGHMNIT LLSDITKQIS R**DYGVLLESA GIALRGLFII DPNGVVKHLS**  **201 VNDLPVGRSV EETLR**LVKAF QFVETHGEVC PANWTPESPT IKPSPTASKE  **251** YFEKVHQ    **Start-End Observed Mr(expt) Mr(calc) Delta Miss Sequence**  **172 - 185 738.9615 1475.9084 1475.7984 0.1101 0 R.DYGVLLESAGIALR.G**  **186 - 197 636.4158 1270.8170 1270.7285 0.0885 0 R.GLFIIDPNGVVK.H**  **198 - 208 603.8791 1205.7436 1205.6517 0.0920 0 K.HLSVNDLPVGR.S**  **209 - 215 417.2480 832.4814 832.4290 0.0524 0 R.SVEETLR.L** |
| 38 | Q3U774 | 56.2 | 5.19 | 96 | 5 | ATP synthase subunit beta, mitochondrial | **1** MLSLVGRVAS ASASGALRGL SPSAALPQAQ LLLRAAPAGV HPARDYAAQA  **51** SAAPKAGTAT GRIVAVIGAV VDVQFDEGLP PILNALEVQG RDSRLVLEVA  **101** QHLGESTVRT IAMDGTEGLV RGQKVLDSGA PIKIPVGPET LGRIMNVIGE  **151** PIDERGPIKT KQFAPIHAEA PEFIEMSVEQ EILVTGIKVV DLLAPYAKGG  **201** KIGLFGGAGV GKTVLIMELI NNVAK**AHGGY SVFAGVGER**T REGNDLYHEM  **251** IESGVINLKD ATSK**VALVYG QMNEPPGAR**A R**VALTGLTVA EYFR**DQEGQD  **301** VLLFIDNIFR FTQAGSEVSA LLGRIPSAVG YQPTLATDMG TMQERITTTK  **351** KGSITSVQAI YVPADDLTDP APATTFAHLD ATTVLSR**AIA ELGIYPAVDP**  **401 LDSTSRIMDP NIVGNEHYDV AR**GVQKILQD YKSLQDIIAI LGMDELSEED  **451** KLTVSRARKI QRFLSQPFQV AEVFTGHMGK LVPLKETIKG FQQILAGEYD  **501** HLPEQAFYMV GPIEEAVAKA DKLAEEHGS    **Start-End Observed Mr(expt) Mr(calc) Delta Miss Sequence**  **226 - 239 469.5956 1405.7650 1405.6739 0.0911 0 K.AHGGYSVFAGVGER.T**  **265 - 279 801.4683 1600.9220 1600.8031 0.1189 0 K.VALVYGQMNEPPGAR.A**  **282 - 294 720.4483 1438.8820 1438.7820 0.1000 0 R.VALTGLTVAEYFR.D**  **388 - 406 994.6011 1987.1876 1987.0262 0.1614 0 R.AIAELGIYPAVDPLDSTSR.I**  **407 - 422 615.0090 1842.0052 1841.8730 0.1322 0 R.IMDPNIVGNEHYDVAR.G** |

**Footnotes:** Mass^1^ = Molecular mass of the protein observed in Mascot search. Mascot score^2^ = >40 indicate identification or extensive homology

(p < 0.05). Peptide matched^3^ = Number of peptides matched with protein in MS/MS query. pI^4^ = Isoeletric point of the protein observed in Mascot search.
